# Book Reviews

**Published:** 1896-08

**Authors:** 


### BOOK REVIEWS.

A Text-Book of Bacteriology. By George M. Sternberg, M.D., LL.D., Surgeon-General U. S. Army, etc., etc. Illustratrated by heliotype and dermo-lithographic plates and 200 engravings. Price, $5.50. New York. William Wood & Co.

For the benefit of students of medicine and others who do not care especially for the detailed descriptions of non-pathogenic bacteria and the extensive bibliography contained in the manual by the same author published in 1892, this text-book has been prepared. It comprises that portion of the manual which is printed in large type, revised to include all important additions to our knowledge of the pathogenic bacteria since the original date of publication.

This text-book of some 700 pages is divided into four parts, the first dealing with classification, morphology and general bacteriology; the second with general biological characters; the third with pathogenic bacteria; and the fourth with saprophytes.

These subjects are dealt with fully and in an interesting and readable manner, while Sternberg's reputation as a bacteriologist is sufficient evidence for the trustworthy nature of this work, which constitutes a book which makes an important addition to the best of America's scientific literature. There is much here described which would be read with benefit by the ordinary busy practitioner; and the book is to be commended to the attention of all who, even without desiring to pose as bacteriologists, yet wish to know something of the life-history of those germs which play such an extremely important rôle in modern pathology, of the method by which they are connected with the diseases which they are believed to produce, and in that way to learn as far as possible what are the present prospects of the physician's ability to destroy them within the human body.

The printing and illustrations are excellent, and do credit to the already high reputation of the publishers.

THE DIAGNOSIS AND TREATMENT OF DISEASES OF THE RECTUM. Being a Practical Treatise on Fistula, Piles, Fissure and Painful Ulcer, Procidentia, Polypus, Stricture, Cancer, etc. By Wm. Allingham, F.R.C.S., Eng., Herbert W. Allingham, F.R.C.S., Eng. Sixth edition. pp. 476. New York, Wm. Wood & Co.

This book needs no words of commendation. It has reached the sixth edition, in which have been made such alterations and additions as seemed advisable in order to bring it up to date. The authors' names are sufficient evidence of the value of the book, for no surgeon has gained a higher reputation in the treatment of the class of diseases with which it deals. We would draw special attention to the section on cancer of the rectum, and especially to the discussion on the advisability of operation. After having during the last ten years seen 850 of these cases, having performed excision 75 times and colotomy 138 times, the authors conclude that if the patient's age does not exceed forty-five years, very little is to be gained by excision, while colotomy seems to prolong life for an extremely short time, and palliative treatment is the right course to pursue. Between the age of forty-five and sixty excision should be attempted when the growth is well in the lower part of the rectum. For patients above sixty excision is extremely favorable in suitable cases. The book is well written and no mistake will be made in recommending it. Marginal notes are used, which greatly facilitate reference. It is well illustrated and beautifully printed.

---

GUIDE MÉDICAL PARISIEN. Published by the "Indépendence, Médicale. pp. 290. Price, 3 fr. 50. A. Malone, Editor, 21 Place de l' école-de-Médecine.

This little book will fill a distinct want felt by all who visit Paris with the intention of seeing the working of its numerous hospitals and cliniques. Each of these receives a short historical description; the names of the various physicians and surgeons, with their chief of staff, the hours of attendance, nature of the work done, with all necessary details, are given.

We notice in the ophthalmic section the absence of the name of Landolt—surely a serious omission, seeing that this celebrated surgeon is one of those visited most frequently and with most result by foreigners. The book deserves, and will no doubt receive, ready sale among native and foreign doctors.

---

THE MULTUM IN PARVO REFERENCE AND DOSE BOOK. By C. Henri Leonard, M.A., M.D., Professor of the Medical and Surgical Diseases of Women, Detroit College of Medicine. Flexible leather, 143 pages, price 75 cents. Detroit. 1896: The Illustrated Medical Journal Company, Publishers.

This is a recent edition of the Dose Book, of which the title page informs us some forty thousand copies have been issued. The present edition is printed on very thin paper, and is bound in red leather, round corners, so as to make it specially light and handy for the pocket; the weight is not two and a half ounces. Besides the doses of some 3,500 preparations being given, it has numerous tables, such as the solubility of chemicals, pronunciation of medical proper names, poisons and their antidotes, incompatibles, tests for urinary deposits, abbreviations, table of fees, etc. It will be found a handy pocket companion.

---

TRANSACTIONS OF THE SOUTHERN SURGICAL AND GYNECOLOGICAL ASSOCIATION. Vol. VIII. Eighth Session, Held at Washington, D. C., Novemver 12, 13, 14, 1895.

We have received a copy of this book, which contains a list of officers, members, the by-laws, with the various addresses and papers read at the meeting. It is well bound and printed.

---

THE fiftieth anniversary number of the *Scientific American*, New York, just out, is a really handsome and valuable publication of 72 pages. It reviews the progress of the past fifty years in the various sciences and industrial arts; and the various articles by the best scientific writers of the day are racily written and richly illustrated. The editors have accomplished the difficult task of presenting a compendium of information that shall be at once historical, technical, and popular. The interest never flags for a moment, and the story of the half-century's growth is in itself a veritable compendium of valuable scientific information for future reference. Price, 10 cents per copy.

